# The interaction of dengue virus capsid protein with negatively charged interfaces drives the *in vitro* assembly of nucleocapsid-like particles

**DOI:** 10.1371/journal.pone.0264643

**Published:** 2022-03-01

**Authors:** Nathane C. Mebus-Antunes, Wellington S. Ferreira, Glauce M. Barbosa, Thais C. Neves-Martins, Gilberto Weissmuller, Fabio C. L. Almeida, Andrea T. Da Poian

**Affiliations:** 1 Instituto de Bioquímica Médica Leopoldo de Meis, Universidade Federal do Rio de Janeiro, Rio de Janeiro, Rio de Janeiro, Brazil; 2 Instituto de Biofísica Carlos Chagas Filho, Universidade Federal do Rio de Janeiro, Rio de Janeiro, Rio de Janeiro, Brazil; 3 Centro Nacional de Biologia Estrutural e Bioimagem, Universidade Federal do Rio de Janeiro, Rio de Janeiro, Rio de Janeiro, Brazil; Universitetet i Bergen, NORWAY

## Abstract

Dengue virus (DENV) causes a major arthropod-borne viral disease, with 2.5 billion people living in risk areas. DENV consists in a 50 nm-diameter enveloped particle in which the surface proteins are arranged with icosahedral symmetry, while information about nucleocapsid (NC) structural organization is lacking. DENV NC is composed of the viral genome, a positive-sense single-stranded RNA, packaged by the capsid (C) protein. Here, we established the conditions for a reproducible *in vitro* assembly of DENV nucleocapsid-like particles (NCLPs) using recombinant DENVC. We analyzed NCLP formation in the absence or presence of oligonucleotides in solution using small angle X-ray scattering, Rayleigh light scattering as well as fluorescence anisotropy, and characterized particle structural properties using atomic force and transmission electron microscopy imaging. The experiments in solution comparing 2-, 5- and 25-mer oligonucleotides established that 2-mer is too small and 5-mer is sufficient for the formation of NCLPs. The assembly process was concentration-dependent and showed a saturation profile, with a stoichiometry of 1:1 (DENVC:oligonucleotide) molar ratio, suggesting an equilibrium involving DENVC dimer and an organized structure compatible with NCLPs. Imaging methods proved that the decrease in concentration to sub-nanomolar concentrations of DENVC allows the formation of regular spherical NCLPs after protein deposition on mica or carbon surfaces, in the presence as well as in the absence of oligonucleotides, in this latter case being surface driven. Altogether, the results suggest that *in vitro* assembly of DENV NCLPs depends on DENVC charge neutralization, which must be a very coordinated process to avoid unspecific aggregation. Our hypothesis is that a specific highly positive spot in DENVC α4-α4’ is the main DENVC-RNA binding site, which is required to be firstly neutralized to allow NC formation.

## Introduction

In recent decades, several pieces of evidence have suggested that viruses and viral capsids are highly dynamic structures [1]. Analytical techniques such as atomic force microscopy (AFM), transmission electron microscopy (TEM), X-ray diffraction, and cryo-electron microscopy (Cryo-EM), have made important contributions to the understanding of the structure and assembly of viral nucleocapsid (NC) [1, 2], an important and complex event in the viral cycle. This event requires optimal conditions, involves intermediate steps and specific time scales, which make its study challenging [1].

Dengue virus (DENV) is a member of the *Flaviviridae* family, which comprises important human pathogens, including Zika virus (ZIKV), West Nile virus (WNV), Yellow fever virus (YFV), Japanese encephalitis virus (JEV), and Tick-borne encephalitis virus (TBEV). DENV structure consists of a spherical particle of 50 nm diameter formed by a lipid bilayer associated with two structural proteins, the membrane (M) and envelope (E) proteins [3, 4], surrounding viral NC, which is composed of the viral genome, a positive-sense single-stranded RNA, packaged by the capsid (C) protein [4].

Despite Cryo-EM studies revealed structural details of mature flavivirus particles, including the organization of E and M proteins on the virus surface, structural information on the NC is still lacking [4–10]. This led flaviviruses’ NC to be considered as an amorphous structure lacking symmetry, unlike alphaviruses’ NC, which have a highly ordered geometry with 240 copies of the capsid protein assembled with a T4 icosahedral symmetry [11–13]. Although Cryo-EM studies of the immature ZIKV particles had recently revealed a partially ordered NC [14, 15], further studies are needed. In addition, little is known about the assembly process of flaviviruses’ NC [16–18]. Viral genome recognition by C protein as well as its interaction with cellular and viral components seem to be very well coordinated, making it difficult to isolate NC from infected cells [19–21].

DENVC is a small (100 residues) and highly basic protein with very particular structural features [22, 23]. It forms homodimers in solution, containing an N-terminal intrinsically disordered region (IDR), followed by 4 inter-twined α-helices connected by short loops, with the tridimensional structure maintained mainly by quaternary contacts [22, 23]. Currently, the most accepted packaging model of the DENV genome is based on an asymmetric charge distribution on the DENVC surface. The presence of 11 apolar residues in the helix α2 generates a hydrophobic cleft in one protein face, while the solvent-exposed region of α4/α4´, rich in basic residues, would act as the RNA binding site [16, 19, 22–24]. However, a more accurate analysis of the electrostatic surface potential of flaviviruses’ C proteins reveals a highly electropositive surface throughout the protein [22]. Thus, considering these particularities, the DENV NC assembly must have a more complex mechanism, not only based on charge asymmetry, with other protein regions being important for assembly.

In this work, we characterized the conditions for an *in vitro* assembly of recombinant DENVC with nucleic acids, a process still very little explored [25]. To our knowledge, this is the first study establishing an efficient *in vitro* assembly of DENV nucleocapsid-like particles (NCLPs) in a systematic way. The assembly protocol established here allowed the visualization of regular particles by AFM and TEM. Using different biophysical techniques, namely small angle X-ray scattering (SAXS), Rayleigh light scattering and fluorescence anisotropy, we characterized the formation NCLPs in solution after incubation of DENVC with small oligonucleotides. Together, these results provided evidence that the formation of stable and complete particles depends on an appropriate surface as well as on the oligonucleotide size length. In addition to contributing to a better understanding of NC assembly, our results also pave the way for future structural investigations.

## Material and methods

### Protein expression and purification

The gene sequence encoding the C protein from DENV serotype 2 (residues 1–100) was cloned into the pET3a by GenScript (Piscataway, NJ, EUA). pET3a-DENVC was transformed into *E*. *coli* BL21-DE3-pLysS. Recombinant DENVC expression was induced in M9 minimal medium, with 0.5 mM isopropyl-D-1-thiogalactopyranoside (IPTG), overnight, at 30 °C. The cells were centrifuged (∼ 5,000 g for 30 min at 4 °C) and the pellets were resuspended in lysis buffer (25 mM HEPES, 0.2 M NaCl, 1 mM EDTA, glycerol 5% (v/v) and protease inhibitor cocktail (P8465, Sigma-Aldrich), pH 7.4). Then, cells were disrupted by ultrasonication. After this step, the lysate was incubated with NaCl at a final concentration of 2 M and left on agitation for 60 min, at 4 °C. Next, the lysate was ultracentrifuged at 70,400 g for 50 min at 4 °C. The supernatant was injected onto a HiTrap Heparin HP column and DENVC was purified using a step gradient with an increasing NaCl concentration (0.5–2 M). Fractions containing DENVC protein were confirmed by 18% SDS-PAGE gel, concentrated, and stored at −20 °C.

### DNA fragments

DNA fragments were purchased from Integrated DNA Technologies, lnc (Coralville, IA, USA). The following single-stranded DNA oligonucleotide (ssDNA) were synthesized: 5’- GG -3’ (2-mer ssDNA); 5’- GGG GG -3’ (5-mer ssDNA) and 5’- TCC ATG GTA GAC AGA GGA TGG GGG A -3’ (25-mer ssDNA). The lyophilized oligonucleotides were resuspended in nuclease-free water to a final concentration of 10 mM. Intermediate stocks of 100 μM were obtained by diluting the initial stock in buffer containing 55 mM NaH_2_PO_4_, 200 mM NaCl, 5 mM EDTA, pH 7.4. The concentrations of 2-, 5- and 25-mer ssDNAs were determined spectrophotometrically using the molar extinction coefficients of 21,600; 51,900 and 259,600 M ^-1^ ·cm ^-1^ at 260 nm, respectively.

### Small angle X-ray scattering (SAXS)

SAXS measurements were performed on beamline SAXS1 at the National Synchrotron Laboratory (Campinas, Brazil). This setup covers a range of momentum transfer of 0.1 < q < 5 nm^-1^ (*q* = 4*π* sin *θ* /*λ*, where 2θ is the scattering angle [26]). SAXS experiments were carried out at 25 ºC in phosphate buffer (55 mM NaH_2_PO_4_, 200 mM NaCl, pH 7.4) using a sample volume of 350 μL in a vacuum tight quartz capillary. The exposure time for each frame was 10 s, and a total of 30 frames were collected for each repeat with intervals of 1 s. The protein concentration used was 60 μM (1.4 mg/mL). For interaction experiments, DENVC was incubated with 2-, 5- and 25-mer ssDNAs at followed DENVC:ssDNA molar ratios: 1:0.1; 1:0.3; 1:0.5; 1:0.7; 1:1, for 2- and 5-mer; and 1:0.01; 1:0.03; 1:0.05; 1:0.07, for 25-mer. All sample measurements were subtracted from the buffer measurement (blank). As the quality of the data depends on the monodisperse and non-aggregated samples, the proteins were centrifuged at 10,000 rpm for 10 min immediately before the incubation. Guinier analysis was carried out using the program PRIMUS/qt, from the ATSAS Suite [27]. The Guinier plot allows the estimation of the radius of gyration (Rg) from the slope in the linear region of a plot of ln (*I* (*q*)) versus *q*^2^, according to Guinier’s relation: I(q)∝exp(−q2Rg23) [26, 28]. Rg was calculated in two ranges: range from 1 to the 10^th^ experimental point (1–10) and range 30–60 (2-mer) or 40–70 (5-mer). These ranges were setup for analyzing the formation of large soluble oligomers and dimeric DENVC in solution, respectively. The calculated Rg for the data series used for the Guinier analysis are summarized in S1 Table.

### Light scattering

Light scattering measurements were performed using Agilent Technologies Care Eclipse equipment. The scattered light was collected at an angle of 90° to the incident light. The samples were excited at 400 nm and the emission was collected from 390 to 410 nm. A slit of 2.5 nm was used for both excitation and emission. Increasing concentrations of DNA oligonucleotides were added to a 5 μM (0.118 mg/mL) protein solution in 400 μL of phosphate buffer (55 mM NaH_2_PO_4_, 200 mM NaCl, 5 mM EDTA, pH 7.4). The oligonucleotide concentration varied from 0 to 15 μM, and experiments were performed in triplicate. After each addition of oligonucleotides, the solution was completely homogenized before fluorescence measurements. Light scattering intensity values were corrected by the dilution factor and normalized as a function of DENVC intensity in absence of oligonucleotides. All sample measurements were subtracted from the buffer (blank). Experimental data from the light scattering measurements of DENVC titrated with increased concentrations of 2-, 5- and 25-mer, as well as their means and standard deviation are summarized in S2 Table.

### Fluorescence anisotropy

The fluorescence anisotropy measurements were carried out using a Cary Eclipse spectrofluorometer (Agilent Technologies, Santa Clara, CA, USA) equipped with a quartz cell of 1.0 cm optical path length, Peltier temperature controller at 25 °C, and excitation and emission polarizers. For investigating the interaction of ssDNA (5-mer and 25-mer) with DENVC, the protein was labeled with fluorescein isothiocyanate (FITC, Sigma-Aldrich). For this, DENVC was dissolved in carbonate-bicarbonate buffer (pH 10) and let to react with FITC (molar ratio FITC:DENVC of 100) for 120 min, in a dark environment at room temperature. Free unreacted fluorophore was separated from the reaction mixture with PD-10 desalting column (GE Healthcare). The labeling efficiency was monitored by absorbance in the regions corresponding to the protein and fluorophore (280 nm for DENVC and 495 nm for FITC). The labeling condition used ensured less than 5% labeling efficiency. This is important since fluorescein binds covalently to protein lysine residues, which would be important for nucleic acid binding and NCLP formation. Fluorescein-labeled DENVC (50 nM solution in phosphate buffer—55 mM NaH_2_PO_4_, 200 mM NaCl and 5 mM EDTA, pH 7.4) was titrated with increasing concentrations of ssDNA (0–0.145 μM). Fluorescence anisotropy was measured exciting at 470 nm and collecting fluorescence emission at 520 nm. A slit of 20 nm was used for both excitation and emission. To ensure that FITC labeling does not change the properties of the protein or impact its interaction with oligonucleotides, similar experiments were performed with non-labeled DENVC using the intrinsic fluorescence of its tryptophan residue (W69). These experiments were collected with excitation wavelength at 280 nm and emission at 346 nm. Each anisotropy measure was an average of 12 accumulations. All experiments were performed in duplicate. Experimental data, their means and standard deviations are summarized in S3 and S4 Tables.

### Atomic Force Microscopy (AFM)

AFM experiments were performed in air at room temperature. First, different DENVC concentrations (15 μM, 15 nM, 1.5 nM or 0.5 nM) in buffer containing 55 mM NaH_2_PO_4_, 200 mM NaCl, 5 mM EDTA (pH 7.4) were applied over the mica surface to set the ideal condition for imaging, avoiding protein overaccumulation on the mica support. For NCLPs visualization, DENVC was prepared at a concentration of 0.5 nM (11.8 ng/mL) and the oligonucleotides were added at a DENVC:ssDNA molar ratios of 2:1 for 5-mer ssDNA, and 20:1 for 25-mer ssDNA. After 10 min of incubation, 200 μL of the sample were uniformly dispersed on the freshly cleaved mica by using a spin coater at speed of 750 rpm for 10 min. Then, three washes with Milli-Q water (200 μL) were performed to remove buffer salts from the mica surface. This step was followed by drying under nitrogen gas. For mica surface charge neutralization experiments, freshly cleaved mica was coated with 3-aminopropyl-trietoxy silane (APTES) to yield an AP-mica surface, as previously described [29]. DENVC at 0.5 nM concentration was incubated for 10 min in the absence or presence of 25-mer ssDNA at 20:1 (DENVC:ssDNA) molar ratio and applied over the treated mica (200 μL of the sample per mica). After 10 min of incubation, samples were washed five times with Milli-Q water (200 μL). The excess liquid was removed with filter paper, followed by drying under nitrogen gas. AFM images were obtained in a Bruker’s Dimension Icon using Tapping Mode^®^. R-TESPA-300 cantilever probe (Bruker, MPP-11120-10) (nominal spring constant 40 N·m^-1^ and nominal tip radius of 8 nm) were used to scan 1 x 1 μm areas of the surface at a resolution of 512 × 512 pixels. Each experiment was performed at least two times. AFM images were processed with Nanoscope Software (Bruker, Karlsruhe, Germany). In the first step of the image processing, polynomial line flattening, and plane fitting were performed. For the analysis of NCLPs’ diameter, the Fiji (ImageJ) software was used to create automated macros that were executed on images to measure the Feret’s diameter of the particles. The tip convolution was corrected considering the nominal tip radius specified by the manufacturer. The correction was made considering the influence of the tip on the edges of each particle as follows: Feret’s diameter—2 (tip radius). Diameter distribution and box plot analysis were performed in GraphPad Prism (GraphPad Software, Inc.).

### Transmission Electron Microscopy (TEM)

For TEM, the *in vitro* assembly was performed as described for the AFM experiments, except that phosphate buffer with 300 mM NaCl was used and an overnight incubation was carried out to allow a better particle stabilization. After incubation, 3.5 μL of the samples (DENVC and DENVC:25-mer) were deposited on carbon-coated 400 mesh copper grids (Electron Microscopy Sciences), previously subjected to glow discharge in Pelco Easiglow ^™^ (at 15 mA and 0.39 mbar air pressure for 25 s). The sample was incubated with the grid for 10 min and then the grid was washed three times with MilliQ water (3.5 μL). Next, the excess liquid was removed with filter paper. Particles assembled *in vitro* were negatively stained with 0.5% sodium phosphotungstate (PTA) for 30 s followed by drying with filter paper. Cytochrome C (Cyt C), a small positively charged (MW = 12 kDa; pI = 9.47) protein, and bovine serum albumin (BSA), a neutral protein, were used as experimental controls in similar TEM experiments (0.5 nM protein in 55 mM phosphate buffer, with 300 mM NaCl, pH 7.4). The grids were analyzed under a FEI Tecnai Spirit operated at 100 kV. Image magnification ranged from 120,000 to 180,000 times. Several independent experiments were carried out Fiji (ImageJ) software was used to measure the Feret’s diameter of the particles. The contrast and brightness were adjusted for maximal visibility of particles. Diameter distribution and box plot analysis were performed in GraphPad Prism (GraphPad Software, Inc.).

### Statistical analysis

Statistical analyses were performed using GraphPad Prism (GraphPad Software, Inc.). Statistical differences in the diameter of NCLPs obtained from the AFM and TEM experiments were analyzed using non-parametric tests; one-way ANOVA with a Kruskal Wallis test and Mann-Whitney U test, respectively. Probability values (p-values) less than 0.05 were considered significant. Box plot and the statistical analysis for the diameters measured for the NCLPs obtained by AFM and TEM are summarized in S5 and S6 Tables.

## Results

### *In vitro* assembly in solution

**Fig 1:**
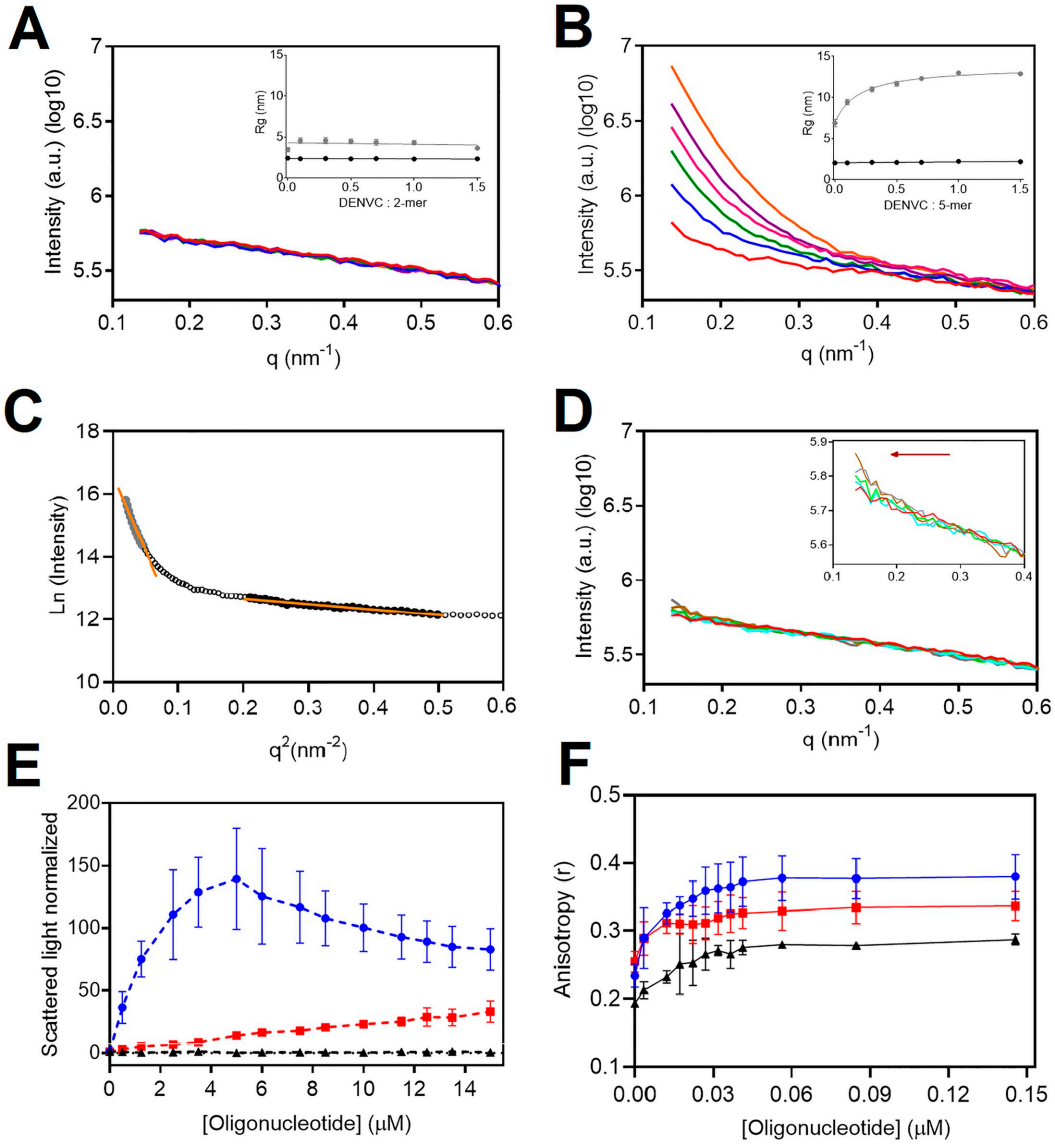
SAXS and light scattering measurements to study oligomerization of DENVC after interaction with ssDNA oligonucleotides. (A and B) SAXS profiles of DENVC (60 μM) obtained with the addition of the oligonucleotide 2-mer and 5-mer, respectively. For A and B, the colors indicate the DENVC:ssDNA molar ratio: free DENVC (red line), 1:0.1 (blue line), 1:0.3 (green line), 1:0.5 (pink line), 1:0.7 (purple line), and 1:1 (orange line). The insert shows the Guinier analysis of the SAXS profiles showing the radius of gyration (Rg) in two ranges. Range from 1 to the 10^th^ experimental point (1–10) corresponds to the SAXS profile region sensitive to the formation of large soluble oligomers (gray circles in A and B). The range 30–60 (2-mer) or 40–70 (5-mer) corresponds to the SAXS region that reports the dimeric DENVC in solution (black circles in A and B). (C) These ranges are exemplified in C. Raw data is represented by white, gray, and black circles. The orange lines represent a linear fit for the lower (gray circles) and upper (black circles) angle region of the data. (D) SAXS profile of DENVC obtained with the addition of 25-mer. The colors indicate the DENVC:ssDNA molar ratio: free DENVC (red line), 1:0.01 (light blue line), 1:0.03 (light green line), 1:0.05 (gray line), 1:0.07 (brown line). The inserted graph is a close-up at low q. The red arrow indicates a possible increase in inflection at low q values which is shifted to the left relative to the 5-mer profile, shown in B. (E) Rayleigh light scattering as a function of ssDNA concentration. DENVC (5 μM) was titrated with 2-mer (black dashed line), 5-mer (red dashed line) and 25-mer (blue dashed line) ssDNA oligonucleotides (0 to 15 μM final concentration). Light scattering intensity values were normalized as a function of DENVC intensity before the addition of oligonucleotides. (F) Fluorescence anisotropy of fluorescein labeled DENVC as a function of ssDNA oligonucleotide concentration. Fluorescein labeled DENVC (50 nM) was titrated with 2-mer (black line), 5-mer (red line) and 25-mer (blue line) ssDNA oligonucleotides (0 to 0.145 μM final concentration). All the experiments were carried out at 25 ºC in phosphate buffer (55 mM NaH_2_PO_4_, 200 mM NaCl, 5 mM EDTA, pH 7.4). The error bars (E and F) represent the standard deviation from duplicate or triplicate measurements. All graphical analyses were performed on GraphPad Prism.

To determine the optimal conditions for *in vitro* assembly of DENVC NCLP in solution as well as the effect of the oligonucleotide size in the particles formed, the recombinant DENVC was incubated with ssDNA of different lengths, and the formation of particles was evaluated by SAXS, Rayleigh light scattering, and fluorescence anisotropy (Fig 1). SAXS measurements of DENVC in the absence of DNA yielded a typical profile of a globular protein. The Guinier analysis indicate a Rg (Rg = 2.05 ± 0.04 nm) compatible with the DENVC dimer, expanded by the presence of the N-terminal IDR (Fig 1A and 1B). The addition of the oligonucleotides promoted a significant increase of the scattered intensity at low values of the scattering angle (q, nm^-1^) (*q* → 0), which indicates the presence of large soluble oligomers. We analyzed the Guinier plot in two regions, as illustrated in Fig 1C. The region 1–10 is sensitive to the formation of large soluble oligomers, while the region 30–60 for 2-mer ssDNA, or 40–70, for 5-mer ssDNA, reports dimeric DENVC in solution without the influence of the soluble oligomers. Note that the Rg obtained from the oligomeric region is constant with the addition of 2-mer ssDNA (Fig 1A, insert) and increased significantly with the addition of 5-mer ssDNA (Fig 1B, insert), reaching a plateau of Rg values of ~ 12.9 nm. The Guinier analysis of the dimer region is constant, maintaining the value of ~ 2.1 nm even upon addition of 2-mer and 5-mer ssDNAs (Fig 1A and 1B, insert). We interpreted these data as suggestive of the presence of dimeric and oligomeric DENVC in solution. The addition of 5-mer ssDNA is significantly more efficient to induce oligomerization than the 2-mer. It is important to mention that for the experiment with 5-mer ssDNA we observed a visual precipitation in molar ratios above 1:1. We also measured the SAXS profiles of DENVC upon addition of the 25-mer ssDNA (Fig 1D). This data also indicated a concentration-dependent oligomerization of DENVC triggered by the addition of the oligonucleotide. However, the data were not so clear to analyze as those obtained for the 5-mer (Fig 1B) because the increase of inflexion at low values of the scattering angle (q, nm^-1^) was shifted to the left, toward smaller values of q. The shift to the left become clear when we note that no effect was observed for q > 0.3 for the 25-mer (Fig 1D) and a significant change was observed for q < 0.5 for the 5-mer (Fig 1B). We believe this result suggest of the formation of soluble oligomers having slightly increased sizes when compared to the oligomers induced by 5-mer. Note that Fig 1D shows the SAXS profiles at low DENVC:25-mer molar ratios. We noticed that at molar ratios higher than 0.1, the curves shifted even more to the left, making them not analyzable with the setup employed. It is also important to mention that visual precipitation is observed at DENVC:25-mer molar ratios higher than 0.1.

To gain more insight on the effect of the ssDNA on DENVC oligomerization, we measured the Rayleigh light scattering upon protein incubation with the oligonucleotides (Fig 1E). The addition of the 5-mer ssDNA led to an increase in light scattering intensity values in a concentration-dependent manner, while this effect was not observed for the 2-mer ssDNA. When using the largest oligonucleotide (25-mer ssDNA), a greater intensity of scattered light was observed even in lower concentrations of oligonucleotide (Fig 1E). This result supports our hypothesis based on the SAXS data (Fig 1D), which showed larger particles (better scatterer) formation in the presence of the 25-mer oligonucleotide.

NCLP formation triggered by 5- and 25-mer was also measured by the increase in the fluorescence anisotropy of fluorescein labeled DENVC (Fig 1F). This experimental approach enables us to work at a 50 nM DENVC concentration, avoiding protein precipitation, as observed in the SAXS and light scattering experiments, which were carried out at the micromolar range. It also allows a better comparison of NCLP formation with 5- and 25-mer oligonucleotides, showing the same stoichiometry of 1:1 (DENVC:oligonucleotide) molar ratio for both, with binding affinities in the nanomolar range. Since fluorescein binds covalently to protein lysine residues that would be important for nucleic acid binding and NCLP formation, we used a protein labelling condition that ensure less than 5% labeling. As an additional control, we measured anisotropy using DENVC intrinsic tryptophan fluorescence, which resulted in a very similar profile of anisotropy increase, although with a much smaller increase in the anisotropy due the small tryptophan fluorescence lifetime (S1 Fig).

### *In-vitro* assembly of NCLPs analyzed by AFM

The results obtained for NCLP assembly in solution showed that the 5-mer and 25-mer oligonucleotides triggered a concentration-dependent DENVC oligomerization. To get more information on DENVC NCLP size, shape, and homogeneity, we performed AFM experiments. In particular, the Tapping Mode^®^ provides topographical information by scanning the sample with an oscillating tip to reduce friction and has been widely employed to study viruses and viral capsids [30, 31].

**Fig 2:**
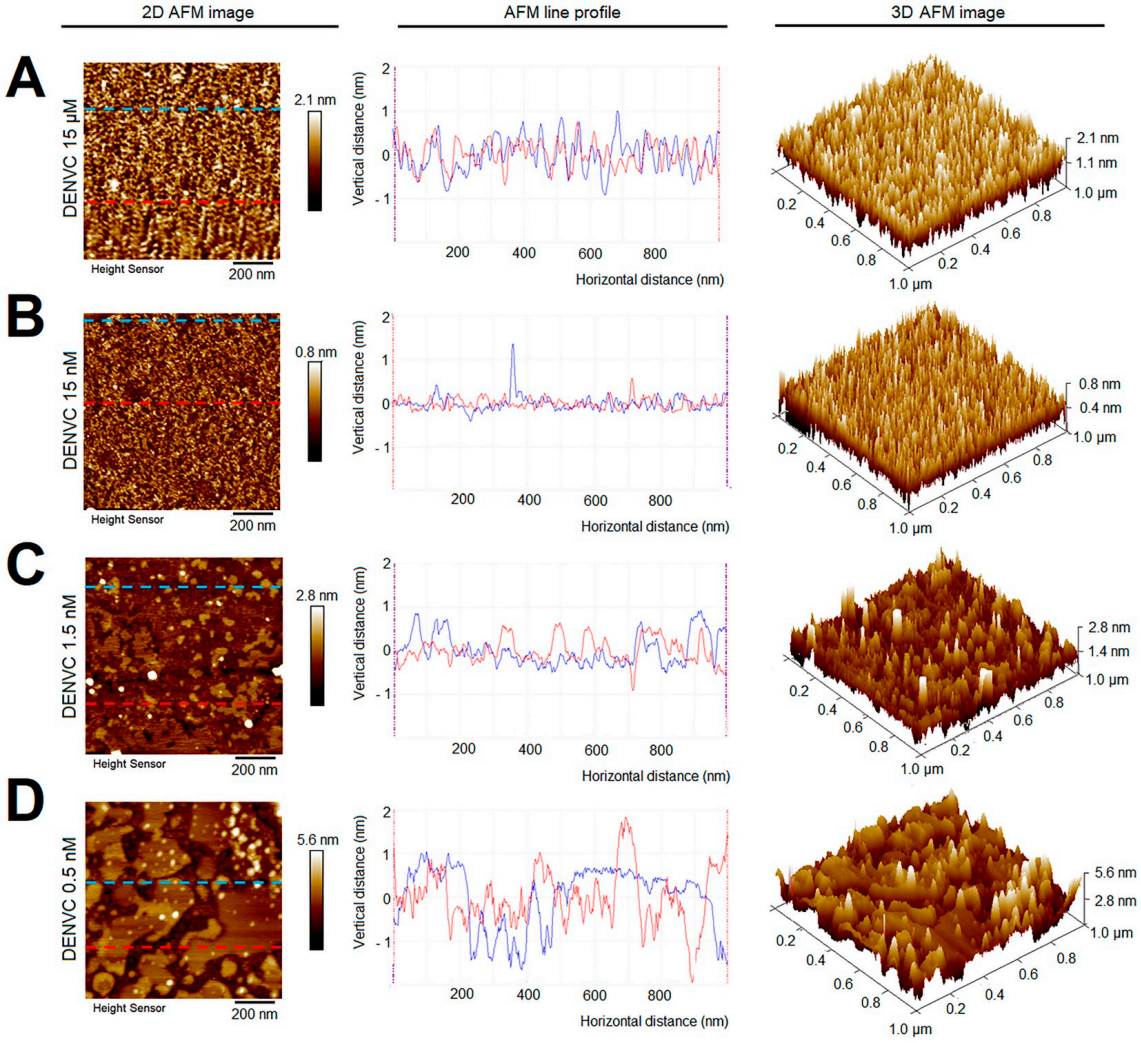
Determination of DENVC concentration for AFM analysis. Representative AFM topographical images (1 μm × 1 μm; *left panels*), line profiles at specific section of the 2D images (*central panels*), and 3D images (*right panels*) of DENVC after dilution in 55 mM NaH_2_PO_4_, 200 mM NaCl, 5 mM EDTA, pH 7.4, to the following concentrations: (A) 15 μM, (B) 15 nM, (C) 1.5 nM and (D) 0.5 nM. The line profile measured in 2D image is shown in the second column. The blue and red dashed lines shown in the 2D images correspond to the sections from where the line profiles were taken. The color scale in the 2D and 3D images indicate the heights in topography. AFM experiments were performed in Tapping Mode^®^, in air, at room temperature. All images and line profiles were acquired with the Nanoscope Analysis software.

The choice of DENVC concentration to be used in assembly reactions was limited by factors inherent to the AFM technique. At higher concentrations, proteins form layers on the mica support, making it difficult to identify individual particles. In addition, at higher concentrations, DENVC presented a strong tendency to form amorphous aggregates during the imaging analyses. Thus, to determine the best protein concentration to perform AFM analysis, we acquired topographic images with systematic protein dilutions in absence of oligonucleotides (Fig 2). Indeed, 2D and 3D AFM images at higher DENVC concentrations showed a thick and rough layer of proteins, which was thinned as the protein was diluted. To provide a more direct comparison of the influence of protein concentration in the protein layer formation, we acquired line profiles along the solid lines drawn in two different regions of the 2D topographical AFM images (red and blue lines in Fig 2, central panels). At low protein concentrations (1.5 nM and 0.5 nM), variations in sample height are observed. These variations are caused by an alternation between regions that contain sample and empty regions, in which we observe the mica surface. Analysis of 2D and 3D AFM images along with the line profiles indicated that the best protein concentration for the assembly reactions was 0.5 nM DENVC (Fig 2D). At this concentration, we were able to obtain a cleaner background that allowed us to evaluate each particle.

**Fig 3:**
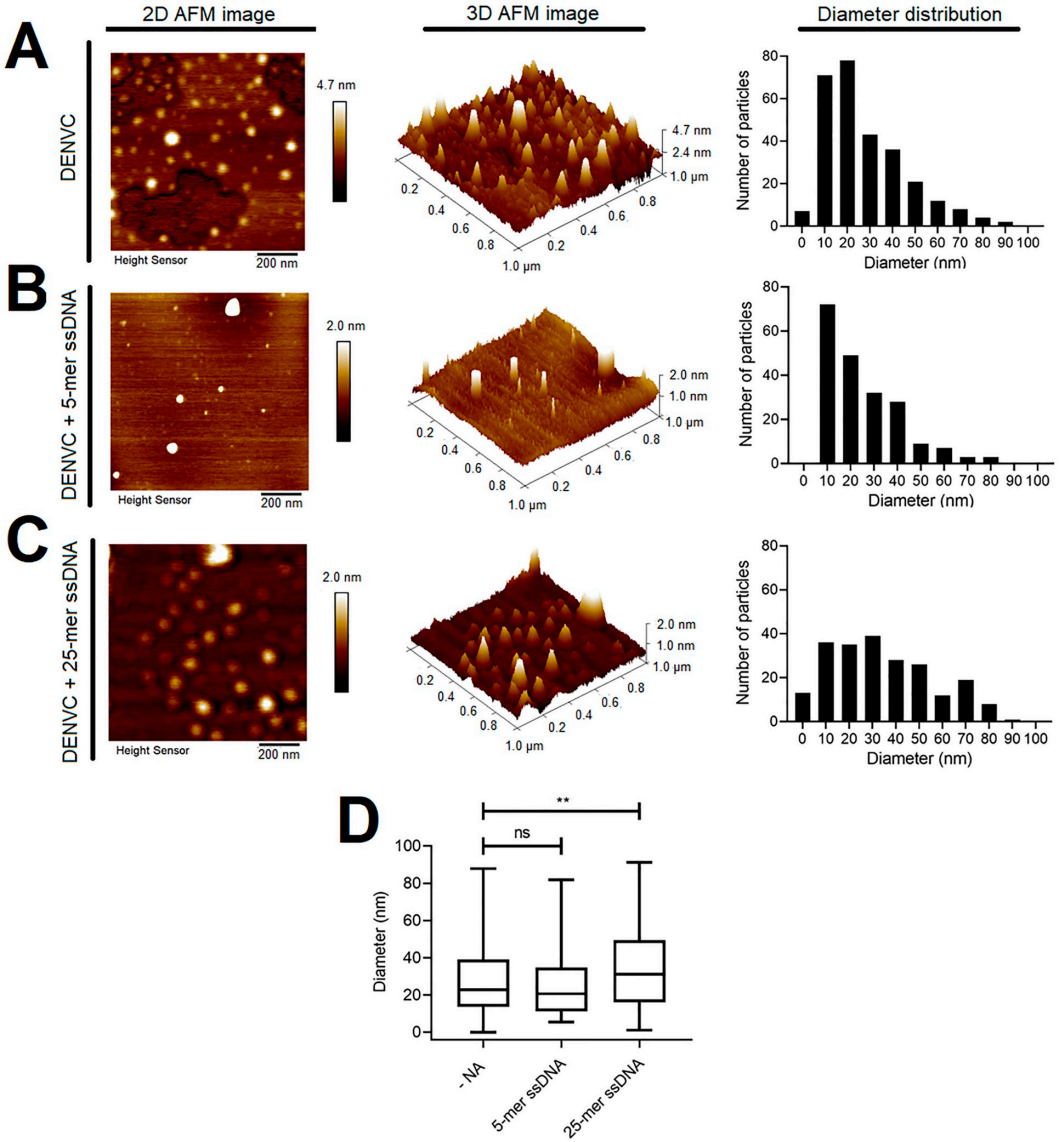
AFM images of recombinant DENVC NCLPs assembled *in vitro*. Representative 2D (*left panels*) and 3D (*central panels*) AFM topographical images (1 × 1 μm), and the Feret’s diameters’ distributions (*right panels*), for the following assembly conditions: (A) DENVC (0.5 nM) alone; (B) DENVC and 5-mer ssDNA, in a 2:1 molar ratio; and (C) DENVC and 25-mer ssDNA, in a 20:1 molar ratio. *In vitro* assembly reaction was performed in 55 mM NaH_2_PO_4_, 200 mM NaCl, 5 mM EDTA (pH:7.4), at room temperature, during 10 min of incubation. The color scale in 2D and 3D images indicates the heights in topography. AFM experiments were performed in Tapping Mode^®^, in air, at room temperature. (D) Box plot graphical representation for the diameters’ distribution obtained for *in vitro* assembly in each condition. The line inside the box denotes the median value (50th percentile) and the whiskers mark the minimum and maximum values. From the minimum value to the beginning of the box, 25% of the data are represented, and from the end of the box to the maximum value, the remaining 25% are represented. The average diameter of particles obtained for DENVC in the absence of oligonucleotides (n = 282), DENVC incubated with 5-mer ssDNA (n = 203) and DENVC incubated with 25-mer ssDNA (n = 216) were 27.6 ± 18.02 nm, 24.82 ± 16.93 nm, and 33.88 ± 21.88 nm, respectively. Statistical analysis was performed using one-way non-parametric ANOVA, followed by Kruskal-Wallis test. Differences were considered significant when P ≤ 0.05 (**, P < 0.01; ns, not significant).

Based on the previous results, we chose 0.5 nM DENVC to perform NCLP assembly experiments. The assembly reactions were carried out with 5- and 25-mer ssDNA, using a DENVC:ssDNA molar ratio of 2:1 and 20:1, respectively. Incubation of DENVC either with the 5-mer or the 25-mer ssDNA resulted in NCLPs showing a regular organization (Fig 3C). The 25-mer-NCLPs appeared to be more symmetrical than the particles obtained after the interaction with the 5-mer (Fig 3B and 3C). Interestingly, we also found particles in absence of oligonucleotide (Fig 3A).

One of the main limitations of the AFM is the lateral broadening effect that affects particle size resolution. This effect is caused by the influence of the probe tip size [32, 33], but other artifacts generated during sample preparation and drying on the mica can also lead to these effects, affecting measurement accuracy. To minimize the lateral broadening effect and perform a quantitative analysis of particle size, we carried out convolution corrections considering the average tip radius value indicated by the cantilever manufacturer. The diameter distribution of NCLPs formed in the absence and presence of 5- and 25-mer ssDNA are represented in Fig 3 (*right panels*). The distribution profile (Fig 3C) formed in the absence and presence of 5-mer ssDNA are similar, with an average diameter of 27.6 ± 18.02 nm and 24.82 ± 16.93 nm for DENVC (n = 282) and DENVC with 5-mer ssDNA (n = 203), respectively, while particles formed in the presence of the 25-mers ssDNA were slightly larger (P < 0.01) (Fig 3D), with an average diameter of 33.88 ± 21.88 nm (n = 216). Box plot representation shows that 50% of NCLPs formed in absence of oligonucleotides or presence of 5-mer ssDNA present 10 to 35 nm-diameter, while in presence of 25-mer ssDNA present 15 to 50 nm-diameter (Fig 3D).

To evaluate whether the highly negatively charged surface of the freshly cleaved mica influenced DENVC NCLP formation, we performed AFM imaging applying the samples after neutralizing (passivating) the AFM surface by coating the cleaved mica with 3-aminopropyl-trietoxy silane (APTES)–AP-mica [29]. The results showed that while for samples prepared in the absence of oligonucleotide only amorphous protein aggregates were observed, particles similar for those observed in AFM images obtained on negatively charges mica were visualized for the samples in the presence of 25-mer ssDNA applied on neutralized surface (S2 Fig). This indicates that DENVC charge neutralization (either by negatively charged surfaces or by nucleic acids) are needed for DENV NCLPs formation.

### NCLPs characterization by TEM

**Fig 4:**
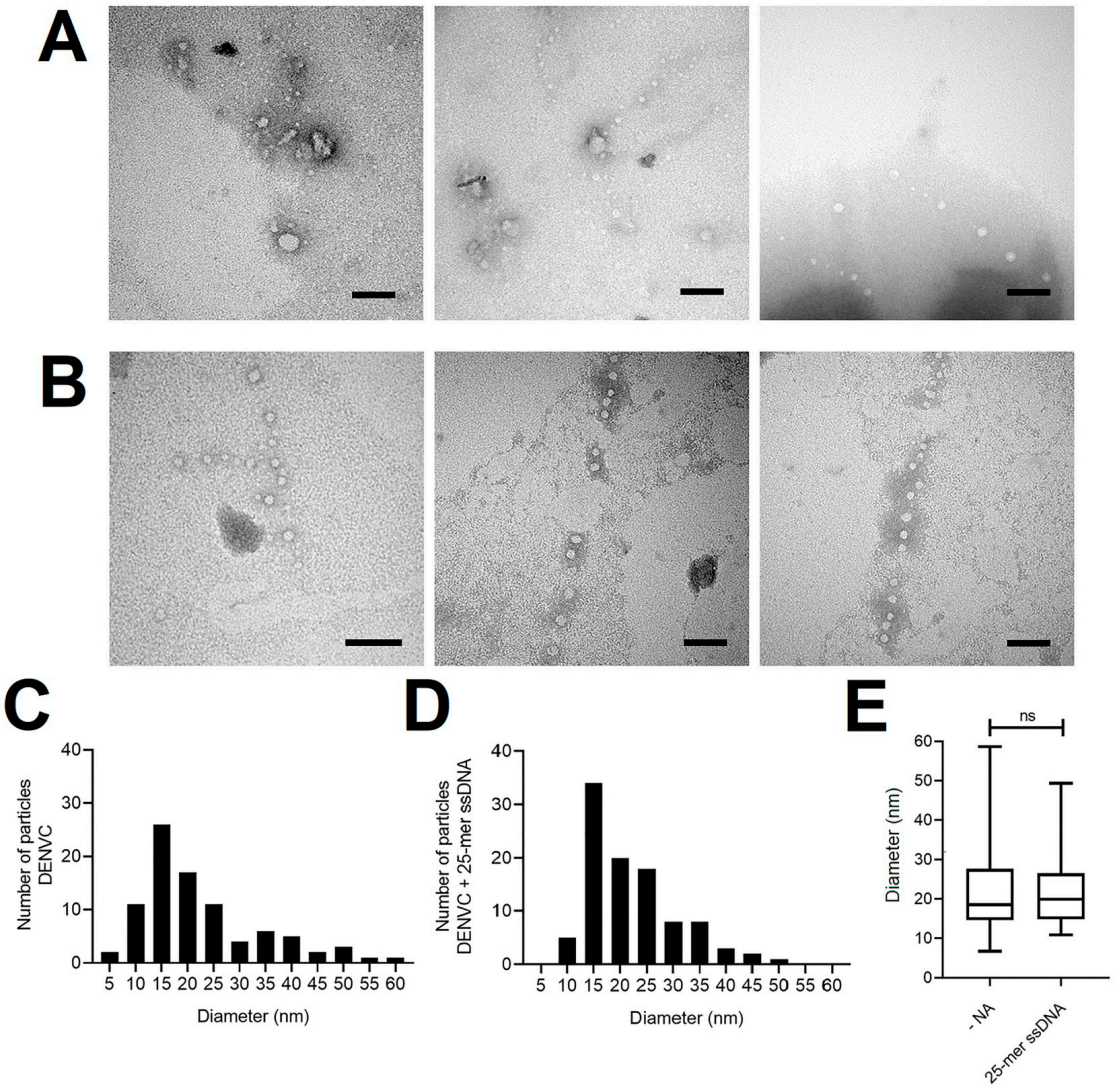
TEM images of recombinant DENVC NCLPs assembled *in vitro*. Representative electron micrographs of NCLPs assembled from DENVC in the (A) absence; and (B) presence of 25-mer ssDNA, in 20:1 molar ratio. *In vitro* assembly reaction of DENVC (0.5 nM) and 25-mer ssDNA (0.025 nM) was performed in buffer containing 55 mM NaH_2_PO_4_, 300 mM NaCl, 5 mM EDTA (pH:7.4), at room temperature and overnight incubation. The grid was stained with 0.5% PTA. Magnification was 120,000 x (except for the first image in B: 150,000 x). All scale bars are 100 nm. The processing of images and measurement of Feret’s diameter was performed using Fiji (ImageJ) software. (C and D) Histograms representing the frequency distribution of particle diameters from DENVC in the (C) absence; and (D) presence of 25-mer ssDNA. (E) Box plot graphical representation for the diameters’ distribution obtained for *in vitro* assembly in each condition. In the diagram, 50% of the data values are represented inside the box and the line indicates the median of the values. The whiskers mark the minimum and maximum values. From the minimum value to the beginning of the box, 25% of the data are represented, and from the end of the box to the maximum value, the remaining 25% are represented. The average diameter of the particles obtained for DENVC (n = 89) and DENVC with 25-mer ssDNA (n = 99) were 22.62 ± 11.59 nm and 22.02 ± 8.75 nm, respectively. Statistical analysis was performed using non-parametric Mann-Whitney U test. Differences were considered significant when P ≤ 0.05 (ns, not significant).

AFM imaging indicated that DENVC forms organized NCLPs *in vitro*. To obtain more information about the DENVC NCLP structure, we used TEM. For this, sample preparation was performed under the same conditions established for AFM and then examined after negative staining (Fig 4). Observation of NCLPs obtained after incubation of DENVC with 25-mer ssDNA showed homogeneous NCLPs with a very regular spherical shape (Fig 4B). It is important to emphasize that NCLPs were not visualized in carbon grids without glow discharge treatment. This observation suggests that the presence of a negatively charged surface is essential to obtain NCLPs under the conditions analyzed.

As observed by AFM, TEM images showed particles in absence of oligonucleotides (Fig 4A). The measured diameters for the particles formed in the presence and absence of 25-mer oligonucleotide were very similar, ranging from 10 to 60 nm, with an average diameter of 22.62 ± 11.59 nm (n = 89) and 22.02 ± 8.75 nm (n = 99) for DENVC in the absence and presence of 25-mer ssDNA, respectively (Fig 4C–4E). In addition, 75% of DENVC NCLPs formed in the presence of 25-mer ssDNA ranged from approximately 10 to 25 nm (Fig 4E). Despite the particles similarity in sizes formed in the absence or presence of oligonucleotides, important morphological differences among them were evidenced by TEM images (Fig 4A and 4B). Although DENVC alone showed a tendency to form particles under these assembly conditions, they do not have the same regularity and organization observed in presence of 25-mer ssDNA. Furthermore, larger and amorphous particles and the presence of aggregates were also observed. The presence of oligonucleotide of an ideal size appears to direct the proteins to organize themselves to form NCLPs more like authentic DENV NC (Fig 4B).

To confirm that surface induced NCLPs self-assembly is a specific property of DENVC, we performed similar TEM experiments under the same conditions as used for DENVC (0.5 nM protein in 55 mM phosphate buffer, with 300 mM NaCl, pH 7.4) using cytochrome C (CytC), a small positively charged (MW = 12 kDa; pI = 9.47) protein as DENVC is, and bovine serum albumin (BSA), a neutral protein usually used as a nonspecific control. In contrast to the results obtained with DENVC, no oligomeric structures or NCLPs were visualized for CytC or BSA (S3 Fig), supporting the hypothesis of the surface-driven self-assembly of DENVC.

## Discussion

Flaviviruses’ capsid proteins play an essential role in interacting with the viral genome, promoting efficient packaging [16, 18, 19]. However, the process of oligomerization and regulation of RNA incorporation during flaviviruses’ nucleocapsid assembly has not yet been described. Although *in vitro* assembly of the recombinant capsid proteins has been well established for alphaviruses [34–36] and hepatitis C virus (HCV) [37], similar systems generated contradictory results for flaviviruses such as DENV [21, 25, 38] and TBEV [39, 40]. Here we described for the first time a successful methodology for systematically follow recombinant DENVC assembly into NCLPs *in vitro*.

**Fig 5:**
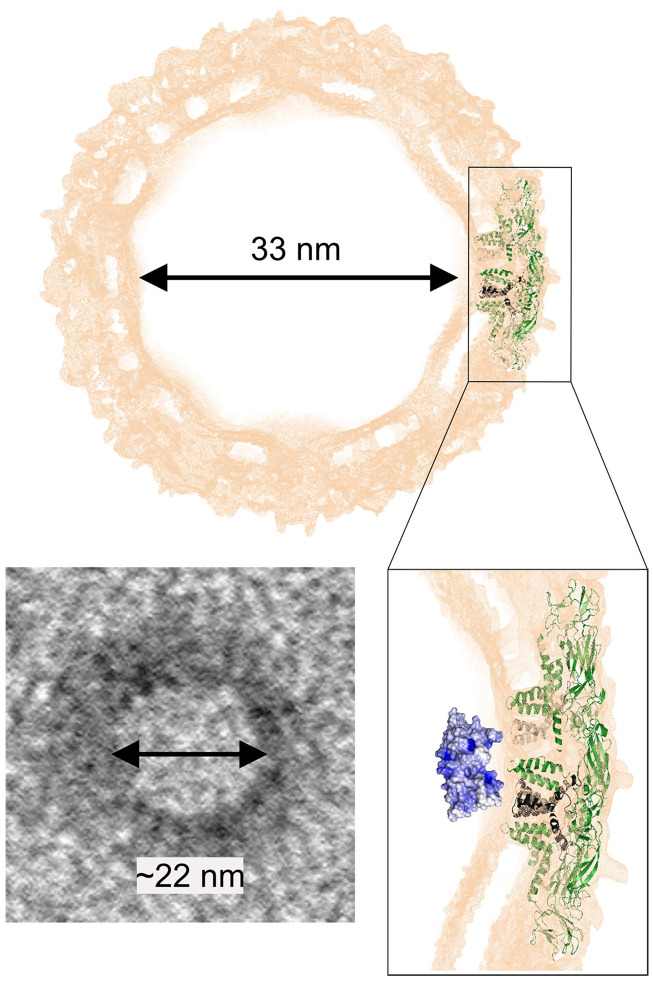
Structure of DENV and schematic model of the assembly of NCLPs. Representation of the external density map (orange) of the cryo-EM structure of mature DENV2 (PDB ID: 3J27), highlighting the position of E (green) and M (dark gray) proteins, and the 33 nm diameter central region, compatible with the NCLPs observed in the present study by TEM, with ~ 22 nm diameter (left lower panel). DENVC (PDB ID: 1R6R) is represented in its hypothetical position in NC (right lower panel). DENVC surface representation was generated in PyMOL, colored according to its electrostatic potential map calculated in APBS software (pH 7.0, 200 mM NaCl, 25 °C) as blue (positive), white (neutral), and red (negative). Note the highly positive spot in α4-α4’ conferred by R85 and K86, which possibly corresponds to the main DENVC-RNA binding site.

Using biophysical techniques, we demonstrated important features of the assembly process in solution, including the nucleic acid size needed to NCLP formation as well as the concentration dependence and the stoichiometry of the process. Comparing the titration of DENVC with oligonucleotides of different sizes (2-, 5- and 25-mer) through different techniques (SAXS, Fig 1A and 1B; Rayleigh light scattering, Fig 1E; and fluorescence anisotropy, Fig 1F), we showed that 2-mer is too small and 5-mer is sufficient for the formation of NCLPs. These data suggest an oligonucleotide size-dependence for particle formation, as already described for other viruses [36, 41]. Our experiments in solution also showed concentration-dependent curves, indicating the formation of large soluble oligomers when using increased concentrations of 5- and 25-mer ssDNA. This stochastic behavior was also confirmed by the anisotropy experiments, revealing a stoichiometry of 1:1 (DENVC:oligonucleotide) molar ratio for both oligonucleotides. Additionally, SAXS studies in which DENVC was incubated with different concentrations of 5-mer oligonucleotide demonstrated a concentration dependence profile with a saturation plateau, indicating an equilibrium involving the DENVC dimer and NCLPs. The Rg of ~12.9 nm obtained by the Guinier analysis is compatible with the sizes of the NCLPs observed by AFM and TEM (average diameter of ~ 22 nm; Figs 3 and 4), as well as with the internal size of the virion (Fig 5). Finally, the fact that NCLPs are formed with the 5-mer oligonucleotide suggests that there is no need of nucleic acid secondary structure to trigger DENVC NCLP assembly. This would be important in the context of virus replication cycle, in which DENVC, as a multifunctional protein, may act either in genome encapsidation or as a chaperone of RNA secondary structures during viral RNA circularization needed for replication [42]. Here, we chose to work with small oligonucleotides, for which chaperone activity of DENVC is not required, simplifying the system.

DENVC shows a high tendency to form amorphous aggregates, which has been reported for the attempts to *in vitro* assembly of NCLPs as well as for isolation of NCs from viral particles purified from cells infected by flaviviruses [38–40]. This occurs probably due to the exposure of hydrophobic surfaces in capsid proteins after interaction with nucleic acids [19, 39]. In the cellular environment, probably the membrane interfaces work as a chaperone for the assembly of organized particles.

Here we surpassed the obstacle of DENVC aggregation in amorphous structures by performing imaging experiments at a very low protein concentration (Fig 2). AFM and TEM imaging showed that the use of sub-nanomolar concentrations of DENVC was essential for the observation of regular and homogeneous NCLPs with minimum aggregation. Accordingly, fluorescence anisotropy experiments also allowed us to work with DENVC at 50 nM concentration, for which we did not observe any aggregation even in excess of DNA.

The NCLPs we observed by TEM are similar in diameter and shape with the previously described DENVC particles assembled *in vitro* [25], in which the authors used mixture of DNA oligonucleotides of 50 bases in a very low proportion (100:1 DENVC:DNA) molar ratio. However, in contrast to the results shown in this previous report [25], or in our experiments performed in solution (Fig 1), we observed the formation of DENVC NCLPs in the absence of nucleic acid when the samples were prepared either for AFM or TEM (Figs 3A and 4A, respectively). We believe that this different result can be explained by the optimized conditions for sample preparations for AFM or TEM imaging, i.e., drying very diluted DENVC solutions over negatively charged surfaces (mica or grid carbon layer, respectively). Accordingly, self-assembly in the absence of nucleic acid has been also reported for HCV [43, 44]. The unique features of flaviviruses’ C proteins, such as its small size and the highly positive surface electrostatic potential [22] (see Fig 5), can further facilitate interaction with the solid charged substrate, driving the self-assembly process. Thus, the presence of the AFM mica surface or the TEM grid carbon surface may have provided a platform for NC formation as it happens in the presence of cellular interfaces [24, 45], helping the NC assembly.

Thus, we hypothesize that DENVC self-assembly is surface-driven, as described for other proteins, including viral proteins [46–49]. In fact, in solution (absence of a surface), DENVC is dimeric and does not oligomerize to large particles, as it was observed in many conditions [21, 23, 50]. In agreement with the surface effect, a previous AFM study demonstrated fast and efficient self-assembly of capsid protein from human immunodeficiency virus (HIV) at very low protein concentration when applied directly onto a negatively charged substrate, showing the need for a surface to drive the process [46, 47]. Ionic strength, pH, temperature, the concentration of molecules, and molecular sequence can affect surface interactions [48, 49]. It is important to mention that we obtained DENVC NCLPs in the absence of oligonucleotide when we used negatively charged surfaces (Figs 3 and 4), but not after surface charge neutralization (S2 Fig), reinforcing that a proper surface can induced NCLP formation rather than amorphous aggregates. In summary, our results suggest that DENV NCLP assembly process depends on a set of factors, such as the low concentration of DENVC, the ionic strength used, the presence of an appropriately sized oligonucleotide, and the presence of a negatively charged surface. The conditions for NCLP formation described here enabled an improved characterization of the concentration dependence for the formation of NCLPs, establishing the presence of an equilibrium between DENVC dimers and NCLPs. Additionally, we believe that flaviviruses’ NC have a highly dynamic nature, which could explain the difficulty observed in obtaining NCLPs the limitation of structural information in cryo-EM studies.

Altogether, our results indicate that, as expected, DENV NC formation requires the neutralization of C protein positive charges through interaction either with nucleic acids or with negatively charged surfaces. However, despite DENVC positive charges have been firstly believed to be concentrated in one face of the protein structure [23], being this asymmetric charge distribution model propagated in the literature, our recent structural analyses measuring long range electrostatic potential revealed a highly electropositive surface throughout the whole protein and not restricted to α4-α4’ (see Fig 5). This is true for DENVC as well as for capsid proteins of other flaviviruses’, such as ZIKV, JEV and WNV [22]. Thus, charge neutralization must be a very coordinated process to avoid unspecific aggregation, explaining the high tendency of DENVC to form amorphous aggregates *in vitro* instead of an organized NC. Our hypothesis is that NC formation depends on neutralization of the highly positive spot conferred by α4-α4’ R85 and K86 (see Fig 5), this being possibly the main DENVC-RNA binding site.

## Supporting information

S1 Fig Anisotropy measurements of non-labeled DENVC titrated with increased concentrations of 25-mer. Non-labeled DENVC (2 μM) was titrated with 25-mer (blue line) ssDNA oligonucleotide (0 to 1.04 μM final concentration). This experiment was carried out at 25 ºC in phosphate buffer (55 mM NaH_2_PO_4_, 200 mM NaCl, 5 mM EDTA, pH 7.4). https://doi.org/10.6084/m9.figshare.19140149.
(TIF)

S2 Fig Effect of neutralizing mica surface on DENVC NCLP formation. DENVC (0.5 nM) samples incubated overnight in the absence (A) or in the presence of 25-mer ssDNA (20:1, DENVC: ssDNA molar ratio) (B) were applied on mica treated with of 3-aminopropyl-triethoxysilane (APTES) for neutralizing surface charges—AP-mica. The color scale in the representative 2D and 3D AFM images (1 x 1 μm), indicate the heights in topography. AFM experiments were performed in Tapping Mode^®^, in air, at room temperature. All images were acquired with the Nanoscope Analysis software. https://doi.org/10.6084/m9.figshare.17838653.v1.
(TIF)

S3 Fig Specificity of DENVC surface-driven NCLPs formation. Representative electron micrographs of (A) DENVC, (B) Cyt C and (C) BSA were incubated overnight, in low concentration (0.5 nM) in buffer containing 55 mM NaH_2_PO_4_, 300 mM NaCl, 5 mM EDTA (pH 7.4), in absence of oligonucleotides, at room temperature. The grids were stained with 0.5% PTA. All scale bars are 100 nm. https://doi.org/10.6084/m9.figshare.17839265.v1.
(TIF)

S1 Table Experimental radius of gyration (Rg) calculated from the Guinier analysis of DENVC: 2- or 5-mer, using the PRIMUS / qt program, from ATSAS Suite. Rg was calculated in two intervals: interval from 1 to 10^th^ experimental point (1–10) and interval 30–60 (2-mer) or 40–70 (5-mer). Values represent the means and errors are the standard deviation between the SAXS data. https://doi.org/10.6084/m9.figshare.17839460.
(DOCX)

S2 Table Experimental data from light scattering measurements of DENVC titrated with increased concentrations of 2-, 5- and 25-mer. Values represent the means and errors are the standard deviation between three independent experiments. https://doi.org/10.6084/m9.figshare.17839679.
(DOCX)

S3 Table Experimental data from anisotropy measurements of fluorescein labeled DENVC titrated with increased concentrations of 2-, 5- and 25-mer. Values represent the means and errors are the standard deviation between two independent experiments. https://doi.org/10.6084/m9.figshare.17839850.
(DOCX)

S4 Table Experimental data from anisotropy measurements of non-labeled DENVC titrated with increased concentrations of 25-mer. The values represent the mean of 12 accumulations and their standard deviations. https://doi.org/10.6084/m9.figshare.19140140.
(DOCX)

S5 Table Box plot and the statistical analysis for the diameters measured for the NCLPs obtained by AFM. https://doi.org/10.6084/m9.figshare.17839988.
(DOCX)

S6 Table Box plot and the statistical analysis for the diameters measured for the NCLPs obtained by TEM. https://doi.org/10.6084/m9.figshare.17840120.
(DOCX)
