# Supplementary figures and images for: The interaction of dengue virus capsid protein with negatively charged interfaces drives the in vitro assembly of nucleocapsid-like particles
Source: PLoS One. 2022 Mar 1;17(3):e0264643. doi: 10.1371/journal.pone.0264643 (PMC8887749; doi:10.1371/journal.pone.0264643)

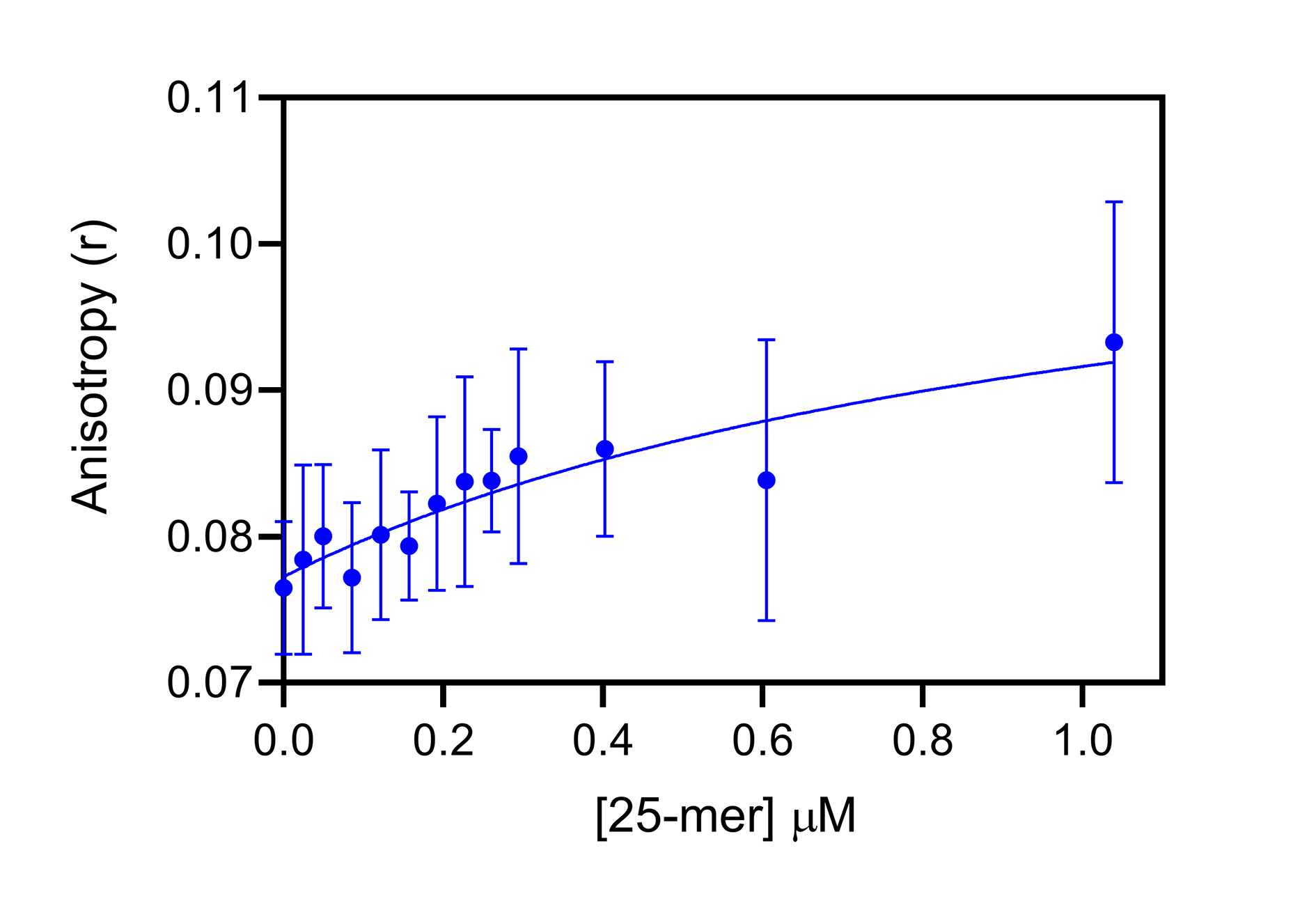

Supplement: S1 Fig — Non-labeled DENVC (2 μM) was titrated with 25-mer (blue line) ssDNA oligonucleotide (0 to 1.04 μM final concentration). This experiment was carried out at 25 ºC in phosphate buffer (55 mM NaH2PO4, 200 mM NaCl, 5 mM EDTA, pH 7.4). https://doi.org/10.6084/m9.figshare.19140149. (TIF) [file pone.0264643.s001.tif]

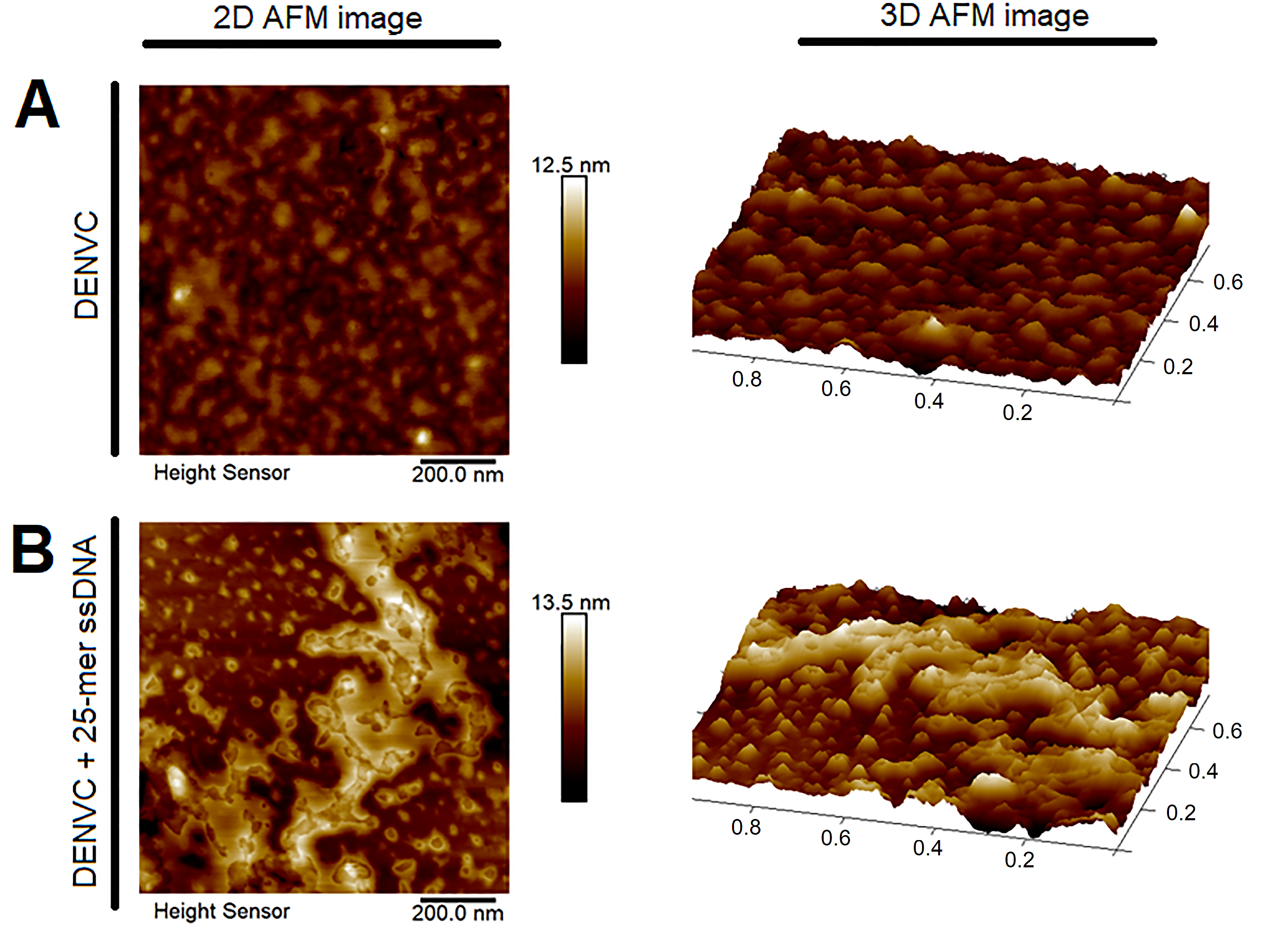

Supplement: S2 Fig — DENVC (0.5 nM) samples incubated overnight in the absence (A) or in the presence of 25-mer ssDNA (20:1, DENVC: ssDNA molar ratio) (B) were applied on mica treated with of 3-aminopropyl-triethoxysilane (APTES) for neutralizing surface charges—AP-mica. The color scale in the representative 2D and 3D AFM images (1 x 1 μm), indicate the heights in topography. AFM experiments were performed in Tapping Mode®, in air, at room temperature. All images were acquired with the Nanoscope Analysis software. https://doi.org/10.6084/m9.figshare.17838653.v1. (TIF) [file pone.0264643.s002.tif]

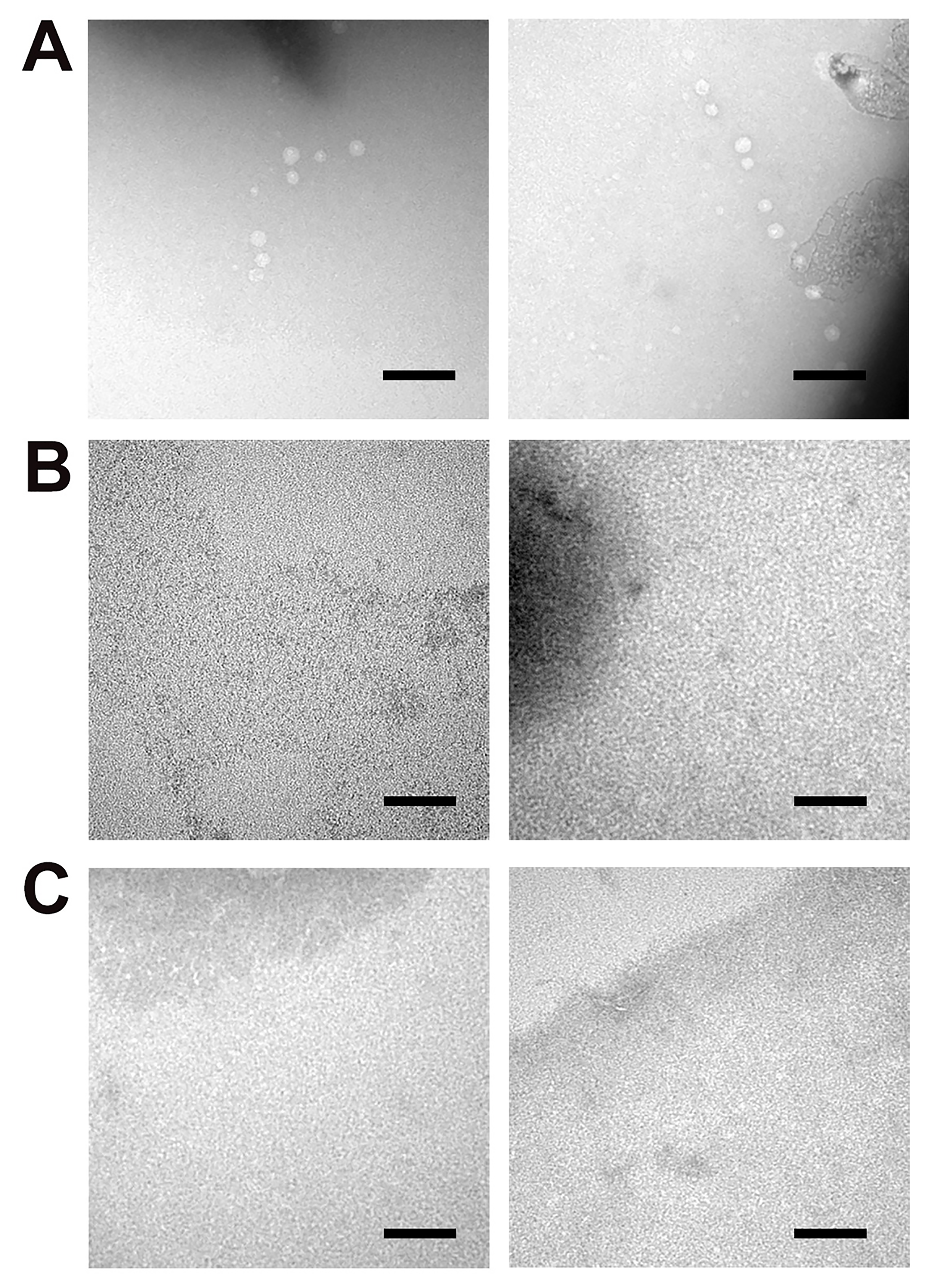

Supplement: S3 Fig — Representative electron micrographs of (A) DENVC, (B) Cyt C and (C) BSA were incubated overnight, in low concentration (0.5 nM) in buffer containing 55 mM NaH2PO4, 300 mM NaCl, 5 mM EDTA (pH 7.4), in absence of oligonucleotides, at room temperature. The grids were stained with 0.5% PTA. All scale bars are 100 nm. https://doi.org/10.6084/m9.figshare.17839265.v1. (TIF) [file pone.0264643.s003.tif]
