# Supplementary material for: The interaction of dengue virus capsid protein with negatively charged interfaces drives the in vitro assembly of nucleocapsid-like particles
Source: PLoS One. 2022 Mar 1;17(3):e0264643. doi: 10.1371/journal.pone.0264643 (PMC8887749; doi:10.1371/journal.pone.0264643)
Supplement: S1 Table — Rg was calculated in two intervals: interval from 1 to 10th experimental point (1–10) and interval 30–60 (2-mer) or 40–70 (5-mer). Values represent the means and errors are the standard deviation between the SAXS data. https://doi.org/10.6084/m9.figshare.17839460. (DOCX) [file pone.0264643.s004.docx]

**S1 Table. Experimental radius of gyration (Rg) calculated from the Guinier analysis of DENVC: 2- or 5-mer, using the PRIMUS / qt program, from ATSAS Suite.** Rg was calculated in two intervals: interval from 1 to 10th experimental point (1-10) and interval 30-60 (2-mer) or 40-70 (5-mer). Values ​​represent the means and errors are the standard deviation between the SAXS data.

|  | Radius of Gyration (Rg) | | | | | | | |
| --- | --- | --- | --- | --- | --- | --- | --- | --- |
|  | **Range 1-10**  **2-mer** | | **Range 30-60**  **2-mer** | | **Range 1-10**  **5-mer** | | **Range 40-70**  **5-mer** | |
| DENVC : ssDNA | **mean** | **SD** | **mean** | **SD** | **mean** | **SD** | **mean** | **SD** |
| 0 | 3.48 | 0.27 | 2.44 | 0.03 | 6.88 | 0.44 | 2.05 | 0.04 |
| 0.1 | 4.57 | 0.35 | 2.36 | 0.04 | 9.44 | 0.33 | 2.05 | 0.04 |
| 0.3 | 4.57 | 0.37 | 2.39 | 0.03 | 11.00 | 0.31 | 2.12 | 0.04 |
| 0.5 | 4.48 | 0.29 | 2.35 | 0.04 | 11.66 | 0.30 | 2.10 | 0.05 |
| 0.7 | 4.36 | 0.35 | 2.38 | 0.04 | 12.31 | 0.26 | 2.12 | 0.04 |
| 1 | 4.32 | 0.27 | 2.33 | 0.03 | 12.99 | 0.24 | 2.24 | 0.03 |
| 1.5 | 3.65 | 0.26 | 2.35 | 0.03 | 12.88 | 0.26 | 2.19 | 0.05 |
