# Supplementary material for: The interaction of dengue virus capsid protein with negatively charged interfaces drives the in vitro assembly of nucleocapsid-like particles
Source: PLoS One. 2022 Mar 1;17(3):e0264643. doi: 10.1371/journal.pone.0264643 (PMC8887749; doi:10.1371/journal.pone.0264643)
Supplement: S2 Table — Values represent the means and errors are the standard deviation between three independent experiments. https://doi.org/10.6084/m9.figshare.17839679. (DOCX) [file pone.0264643.s005.docx]

**S2 Table. Experimental data from light scattering measurements of DENVC titrated with increased concentrations of 2-, 5- and 25-mer.** Values ​​represent the means and errors are the standard deviation between three independent experiments.

| [ssDNA] | 2-mer | | | | | 5-mer | | | | | 25-mer | | | | |
| --- | --- | --- | --- | --- | --- | --- | --- | --- | --- | --- | --- | --- | --- | --- | --- |
|  | **Scattered light normalized** | | | **Mean** | **SD** | **Scattered light normalized** | | | **mean** | **SD** | **Scattered light normalized** | | | **mean** | **SD** |
| 0.0 | 1.000 | 1.000 | 1.000 | 1.000 | 0.000 | 1.000 | 1.000 | 1.000 | 1.000 | 0.000 | 1.000 | 1.000 | 1.000 | 1.000 | 0.000 |
| 0.5 | 0.537 | 0.548 | 1.796 | 0.960 | 0.723 | 2.488 | 4.132 | 2.433 | 3.018 | 0.965 | 40.233 | 46.827 | 22.294 | 36.451 | 12.696 |
| 1.3 | 0.475 | 0.651 | -0.067 | 0.353 | 0.374 | 5.789 | 2.823 | 6.985 | 5.199 | 2.143 | 83.144 | 83.804 | 58.775 | 75.241 | 14.264 |
| 2.5 | 0.561 | 0.685 | 0.814 | 0.687 | 0.126 | 7.771 | 7.426 | 5.088 | 6.762 | 1.460 | 104.134 | 149.635 | 78.371 | 110.714 | 36.085 |
| 3.5 | 1.233 | 0.803 | 1.775 | 1.270 | 0.487 | 9.763 | 8.176 | 7.709 | 8.549 | 1.077 | 144.872 | 144.776 | 96.560 | 128.736 | 27.865 |
| 5.0 | 0.372 | 0.615 | -0.360 | 0.209 | 0.508 | 14.054 | 14.344 | 13.621 | 14.006 | 0.364 | 176.620 | 145.427 | 96.256 | 139.435 | 40.516 |
| 6.0 | 1.166 | 0.580 | -0.215 | 0.510 | 0.693 | 15.657 | 18.189 | 15.394 | 16.413 | 1.543 | 162.224 | 128.169 | 85.703 | 125.365 | 38.337 |
| 7.5 | 0.907 | 1.155 | -0.186 | 0.625 | 0.714 | 17.804 | 18.826 | 16.873 | 17.834 | 0.977 | 142.544 | 122.014 | 85.630 | 116.729 | 28.822 |
| 8.5 | 0.805 | 0.899 | -0.233 | 0.491 | 0.628 | 20.179 | 20.821 | 20.732 | 20.578 | 0.348 | 125.644 | 114.515 | 83.361 | 107.840 | 21.917 |
| 10.0 | 1.286 | 0.351 | -0.361 | 0.426 | 0.826 | 21.959 | 25.862 | 21.409 | 23.077 | 2.428 | 111.791 | 111.048 | 78.328 | 100.389 | 19.109 |
| 11.5 | 1.276 | 0.985 | 0.215 | 0.825 | 0.548 | 25.670 | 28.316 | 21.302 | 25.096 | 3.542 | 101.962 | 104.026 | 72.371 | 92.786 | 17.710 |
| 12.5 | 1.488 | 0.804 | -0.326 | 0.655 | 0.916 | 28.494 | 36.310 | 21.728 | 28.844 | 7.297 | 93.931 | 103.062 | 70.692 | 89.228 | 16.689 |
| 13.5 | 0.783 | 0.488 | 2.459 | 1.244 | 1.063 | 29.392 | 34.708 | 21.516 | 28.539 | 6.637 | 89.784 | 98.495 | 66.720 | 85.000 | 16.419 |
| 15.0 | 1.057 | 0.733 | -0.117 | 0.558 | 0.607 | 34.296 | 40.959 | 23.995 | 33.083 | 8.547 | 86.676 | 97.245 | 64.493 | 82.804 | 16.716 |
