# Supplementary material for: The interaction of dengue virus capsid protein with negatively charged interfaces drives the in vitro assembly of nucleocapsid-like particles
Source: PLoS One. 2022 Mar 1;17(3):e0264643. doi: 10.1371/journal.pone.0264643 (PMC8887749; doi:10.1371/journal.pone.0264643)
Supplement: S3 Table — Values represent the means and errors are the standard deviation between two independent experiments. https://doi.org/10.6084/m9.figshare.17839850. (DOCX) [file pone.0264643.s006.docx]

**S3 Table. Experimental data from anisotropy measurements of fluorescein labeled DENVC titrated with increased concentrations of 2-, 5- and 25-mer.** Values ​​represent the means and errors are the standard deviation between two independent experiments.

| [ssDNA] | 2-mer | | | | 5-mer | | | | 25-mer | | | |
| --- | --- | --- | --- | --- | --- | --- | --- | --- | --- | --- | --- | --- |
|  | r | | Mean | SD | r | | Mean | SD | r | | Mean | SD |
| 0.0000 | 0.1935 | 0.1938 | 0.1937 | 0.0002 | 0.2657 | 0.2442 | 0.2549 | 0.0152 | 0.2462 | 0.2220 | 0.2341 | 0.0171 |
| 0.0035 | 0.2215 | 0.2039 | 0.2127 | 0.0124 | 0.3065 | 0.2714 | 0.2889 | 0.0248 | 0.3211 | 0.2582 | 0.2896 | 0.0445 |
| 0.0120 | 0.2258 | 0.2394 | 0.2326 | 0.0096 | 0.3227 | 0.3005 | 0.3116 | 0.0157 | 0.3367 | 0.3149 | 0.3258 | 0.0154 |
| 0.0171 | 0.2194 | 0.2826 | 0.2510 | 0.0447 | 0.3208 | 0.2992 | 0.3100 | 0.0152 | 0.3467 | 0.3280 | 0.3374 | 0.0132 |
| 0.0220 | 0.2293 | 0.2772 | 0.2533 | 0.0339 | 0.3292 | 0.2899 | 0.3096 | 0.0278 | 0.3657 | 0.3291 | 0.3474 | 0.0259 |
| 0.0269 | 0.2496 | 0.2821 | 0.2659 | 0.0230 | 0.3279 | 0.2943 | 0.3111 | 0.0237 | 0.3842 | 0.3349 | 0.3596 | 0.0348 |
| 0.0317 | 0.2659 | 0.2767 | 0.2713 | 0.0077 | 0.3361 | 0.3016 | 0.3188 | 0.0244 | 0.3882 | 0.3373 | 0.3628 | 0.0360 |
| 0.0365 | 0.2511 | 0.2805 | 0.2658 | 0.0208 | 0.3449 | 0.3057 | 0.3253 | 0.0277 | 0.3907 | 0.3388 | 0.3647 | 0.0367 |
| 0.0412 | 0.2679 | 0.2835 | 0.2757 | 0.0110 | 0.3426 | 0.3099 | 0.3262 | 0.0231 | 0.3988 | 0.3465 | 0.3727 | 0.0370 |
| 0.0563 | 0.2778 | 0.2824 | 0.2801 | 0.0032 | 0.3496 | 0.3090 | 0.3293 | 0.0287 | 0.4012 | 0.3546 | 0.3779 | 0.0330 |
| 0.0846 | 0.2770 | 0.2805 | 0.2787 | 0.0024 | 0.3516 | 0.3176 | 0.3346 | 0.0241 | 0.3980 | 0.3563 | 0.3772 | 0.0295 |
| 0.1455 | 0.2932 | 0.2821 | 0.2877 | 0.0078 | 0.3522 | 0.3214 | 0.3368 | 0.0217 | 0.4033 | 0.3564 | 0.3799 | 0.0332 |
