# Supplementary material for: The interaction of dengue virus capsid protein with negatively charged interfaces drives the in vitro assembly of nucleocapsid-like particles
Source: PLoS One. 2022 Mar 1;17(3):e0264643. doi: 10.1371/journal.pone.0264643 (PMC8887749; doi:10.1371/journal.pone.0264643)
Supplement: S4 Table — The values represent the mean of 12 accumulations and their standard deviations. https://doi.org/10.6084/m9.figshare.19140140. (DOCX) [file pone.0264643.s007.docx]

**S4 Table. Experimental data from anisotropy measurements of non-labeled DENVC titrated with increased concentrations of 25-mer.** The values represent the mean of 12 accumulations and their standard deviations.

| [25-mer] | Anisotropy (r) | |
| --- | --- | --- |
|  | Mean SD | |
| 0.000 | 0.077 | 0.005 |
| 0.025 | 0.078 | 0.006 |
| 0.050 | 0.080 | 0.005 |
| 0.086 | 0.077 | 0.005 |
| 0.122 | 0.080 | 0.006 |
| 0.157 | 0.079 | 0.004 |
| 0.192 | 0.082 | 0.006 |
| 0.227 | 0.084 | 0.007 |
| 0.261 | 0.084 | 0.003 |
| 0.294 | 0.085 | 0.007 |
| 0.402 | 0.086 | 0.006 |
| 0.604 | 0.084 | 0.010 |
| 1.040 | 0.093 | 0.010 |
