# Supplementary material for: The interaction of dengue virus capsid protein with negatively charged interfaces drives the in vitro assembly of nucleocapsid-like particles
Source: PLoS One. 2022 Mar 1;17(3):e0264643. doi: 10.1371/journal.pone.0264643 (PMC8887749; doi:10.1371/journal.pone.0264643)
Supplement: S5 Table — https://doi.org/10.6084/m9.figshare.17839988. (DOCX) [file pone.0264643.s008.docx]

**S5 Table. Box plot and the statistical analysis for the diameters measured for the NCLPs obtained by AFM.**

|  | DENVC | 5-mer ssDNA | 25-mer ssDNA |
| --- | --- | --- | --- |
| Total number of values | 282 | 203 | 216 |
|  |  |  |  |
| Minimum | 0.015 | 5.519 | 1.091 |
| 25% Percentile | 13.75175 | 11.22 | 16.14 |
| Median | 22.794 | 20.646 | 30.8035 |
| 75% Percentile | 39.025 | 34.818 | 48.78425 |
| Maximum | 87.873 | 81.924 | 84.314 |
|  |  |  |  |
| Mean | 27.59217021 | 24.82342857 | 33.88801389 |
| Std. Deviation | 18.02155468 | 16.93669464 | 21.8884772 |
| Std. Error of Mean | 1.073167964 | 1.188722925 | 1.489322233 |
|  |  |  |  |
| Lower 95% CI of mean | 25.47970122 | 22.47953159 | 30.95247172 |
| Upper 95% CI of mean | 29.7046392 | 27.16732555 | 36.82355606 |
|  |  |  |  |
| Statistical method used | One-way non-parametric ANOVA/Kruskal Wallis: compare selected pairs | | |
| P value | **, P < 0.01 (25-mer ssDNA vs. DENVC) | | |
