# Supplementary material for: The interaction of dengue virus capsid protein with negatively charged interfaces drives the in vitro assembly of nucleocapsid-like particles
Source: PLoS One. 2022 Mar 1;17(3):e0264643. doi: 10.1371/journal.pone.0264643 (PMC8887749; doi:10.1371/journal.pone.0264643)
Supplement: S6 Table — https://doi.org/10.6084/m9.figshare.17840120. (DOCX) [file pone.0264643.s009.docx]

**S6 Table. Box plot and the statistical analysis for the diameters measured for the NCLPs obtained by TEM**

|  | DENVC | 25-mer ssDNA |
| --- | --- | --- |
| Total number of values | 89 | 99 |
|  |  |  |
| Minimum | 6.701 | 10.889 |
| 25% Percentile | 14.6245 | 14.818 |
| Median | 18.524 | 19.877 |
| 75% Percentile | 27.62425 | 26.4955 |
| Maximum | 58.632 | 49.3755 |
|  |  |  |
| Mean | 22.62633146 | 22.01834848 |
| Std. Deviation | 11.59292849 | 8.752579997 |
| Std. Error of Mean | 1.228847962 | 0.879667388 |
|  |  |  |
| Lower 95% CI of mean | 20.18425436 | 20.27267718 |
| Upper 95% CI of mean | 25.06840856 | 23.76401979 |
|  |  |  |
| Statistical method used | Mann Whitney U test | |
| P value | not significant (ns), P > 0.05 | |
